# Multiple Pseudo-Placentational Endometrial Hyperplasia (PEH) as a Rare Uterine Disorder in the Bitch

**DOI:** 10.3390/ani15040479

**Published:** 2025-02-08

**Authors:** Andrzej Łobaczewski, Rafał Sapierzyński, Sławomir Giziński, Ewa Kautz-Wasilewska, Anna Jaśkiewicz, Olga Szaluś-Jordanow, Zofia Nowek, Agata Moroz-Fik, Michał Grzegorczyk, Tadeusz Frymus

**Affiliations:** 1 Veterinary Clinic Auxilium, Królewska Str. 64, 05-822 Milanówek, Poland; alobaczewski007@gmail.com (A.Ł.); chatanga@gmail.com (A.J.); 2 Department of Pathology and Veterinary Diagnostic, Institute of Veterinary Medicine, Warsaw University of Life Sciences-SGGW, Nowoursynowska Str. 159, 02-776 Warsaw, Poland; rafal_sapierzynski@sggw.edu.pl; 3 Department of Large Animal Diseases and Clinic, Warsaw University of Life Sciences-SGGW, Nowoursynowska 100, 02-797 Warsaw, Poland; slawomir_gizinski@sggw.edu.pl (S.G.); ewa_kautz@sggw.edu.pl (E.K.-W.); 4 Department of Small Animal Diseases with Clinic, Institute of Veterinary Medicine, Warsaw University of Life Sciences-SGGW, Nowoursynowska Str. 159c, 02-776 Warsaw, Poland; tadeusz_frymus@sggw.edu.pl; 5 Division of Veterinary Epidemiology and Economics, Institute of Veterinary Medicine, Warsaw University of Life Sciences-SGGW, Nowoursynowska Str. 159c, 02-776 Warsaw, Poland; zofia_nowek@sggw.edu.pl (Z.N.); agata_moroz_fik@sggw.edu.pl (A.M.-F.); 6 Department of Descriptive and Clinical Anatomy, Medical University of Warsaw, Chałubińskiego 5, 02-004 Warsaw, Poland; grzegorczyk.m.s@gmail.com

**Keywords:** dog, placenta, pseudo-placentational endometrial hyperplasia, uterine, deciduoma

## Abstract

The reproductive cycle in domestic dogs involves prolonged phases of hormonal activity, making the endometrial tissue vulnerable to degenerative changes. One common result of these changes is cystic endometrial hyperplasia (CEH), which often leads to pyometra. Another, though rarer, condition is pseudo-placentational endometrial hyperplasia (PEH), characterized by abnormal proliferation of the endometrial glands and stroma, forming placental-like structures without the presence of pregnancy. Case presentation: An 8-year-old female Golden Retriever, healthy and intact, was presented for a routine ultrasound examination to confirm pregnancy after natural breeding 25 days earlier. Two previous pregnancies of this bitch have resulted in small but live litters. The ultrasound examination revealed multiple bilateral thickenings of the uterine horns, with ampullary-shaped enlargements measuring approximately 2.5 × 3.0 cm 25 days after breeding. The endometrium was conspicuously thickened, hypoechogenic, and either homogeneous or with scattered hyperechoic markings and faint striations. The mucosal surface ranged from regular to slightly irregular, and a small amount of hyperechoic condensed fluid was present in the lumen of the horns. No embryos were observed. Based on these findings, two primary diagnoses were considered: PEH or embryonic resorption sites, with a possible segmental uterine inflammation. A diagnosis suggestive of PEH was established through histopathological examination of the uterus after ovariohysterectomy, supported by consistent ultrasound findings that showed no progression or changes over time.

## Simple Summary

The reproductive system of female dogs is prone to various conditions due to prolonged hormonal activity. One such condition is pseudo-placentational endometrial hyperplasia (PEH). In this study, we describe the case of an 8-year-old Golden Retriever who was was referred to the clinic for a routine ultrasound 25 days after natural breeding. Although the dog had previously given birth to live puppies, the ultrasound revealed unusual uterus wall thickenings. This was not the typical appearance of embryonic vesicles, as neither an embryo nor a gestational sac was visualized. The appearance of the uterus on ultrasound examination may resemble or suggest multiple resorption vesicles. Serial ultrasound examinations did not show any remodeling over time. After surgically removing the uterus and ovaries, histopathological examination along with the previous ultrasound examinations, which showed no changes over time, confirmed the diagnosis of PEH. This knowledge benefits veterinarians and pet owners, helping to improve reproductive healthcare for dogs. The study describes a case of multiple PEH in a dog after natural mating.

## 1. Introduction

The reproductive cycle in carnivores is a unique evolutionary strategy characterized by prolonged estrogenic, progestogenic, and prolactin phases. Within this complex hormonal background, the endometrium of the uterus acts as the principal effector organ for both ovarian steroids and pituitary hormones [1,2,3]. In healthy canine pregnancies, the uterus undergoes significant enlargement to accommodate developing embryos and fetuses. Following parturition, or embryonic resorption, occurring between days 19 and 35, or abortions, the uterus typically returns to its pre-pregnancy state through a process known as uterine involution. This process involves a reduction in uterine size and wall thickness. After embryonic death, the process involves detachment of the placenta from the uterine wall and the resorption of tissues, ultimately leading to the complete loss of all embryonic structures [4,5].

In certain circumstances, especially in older individuals, the repetitive hormonal changes through the diestrus cycle make the endometrial tissue susceptible to degenerative changes. One of the most common conditions affecting domestic dogs in this context is cystic endometrial hyperplasia (CEH), a well-known metropathy. CEH is characterized by a persistent thickening of the uterine lining due to the formation of cystic structures within the endometrium. Microscopically, CEH is characterized by distinct structural changes in the uterine horns, including significant endometrial tissue proliferation and cysts on the endometrial surface. Additionally, the marked proliferation of the endometrial epithelial lining and transformation of the glandular epithelium into the secretory phase is observed. It is important to emphasize that cystic endometrial hyperplasia can progress to the development of pyometra; however, this progression is not inevitable [6]. This condition is often associated with repeated exposure to progesterone during diestrus, leading to pathological changes that impair the uterus’s ability to revert to its normal state. Consequently, the uterus remains enlarged and does not undergo typical involution. In contrast, pseudo-placentational endometrial hyperplasia (PEH) is a rare but equally perplexing condition. In PEH, the endometrium proliferates in a highly organized manner and histopathologically mimics placentation sites of normal pregnancy [7]. This pathological state has been observed since the 20th century as a side effect of experimental physical contraception in dogs. Nomura et al. confirmed that the implantation of foreign bodies led to deciduoma-like lesions in the endometrium [8,9,10]. There is a possibility that PEH may be induced by early embryo resorption, as cases of concurrent PEH and the successful completion of a healthy pregnancy have been reported [11]. Cases of PEH have also been reported in bitches following natural mating in the absence of confirmed pregnancy [12,13]. It is also suspected that PEH could be associated with high progesterone levels [14].

In both CEH and PEH, excessive endometrial secretions may facilitate the development of bacterial infections and subsequent inflammation (endometritis), potentially progressing to pyometra [15,16].

Therefore, while the histopathological examination is essential for diagnosis, the persistence of lesions is also a crucial diagnostic factor [11,12,13].

## 2. Materials and Methods

An 8-year-old, clinically healthy Golden Retriever female was presented for a routine ultrasound examination to ascertain pregnancy subsequent to natural mating 25 days earlier.

The bitch had twice undergone successful breeding, three years and one year prior to the current examination, resulting in the birth of healthy offspring through natural parturition. Approximately four weeks before visiting the veterinary clinic, serum progesterone levels were assessed to determine the optimal time for breeding.

The hormonal testing was conducted independently by the owner. Therefore, neither general blood tests nor vaginal cytology was performed, and the gynecological examination was minimized. The progesterone level was measured using chemiluminescent enzyme immunoassay (IMMULITE^®^ 2000, Siemens Healthcare Diagnostics Inc., Deerfield, FL, USA) on days 5, 3, and 1 prior to mating, with results of 1.00 ng/mL, 2.00 ng/mL, and 18.73 ng/mL, respectively. The bitch was mated the day after the final measurement.

### Clinical Examination

The dog’s clinical examination did not show any abnormalities. The initial ultrasonographic evaluation carried out on the 25th day after mating, by a general practitioner, revealed no signs of pregnancy. However, ampulla-like, oval, hypoechoic thickening of the uterine horns was observed, with each horn exhibiting two formations, accompanied by a minor amount of fluid content within the lumen. Embryonic resorption with the presence of resorption ampullae was initially suspected. Due to suspected inflammatory component in uterine horns, ceftiofur 2.2 mg/kg s.c. (Excenel^®^, Zoetis, Warsaw, Poland) was applied for seven days. The clinical status of the patient remained normal, and no vaginal discharge was noted.

**Figure 1:**
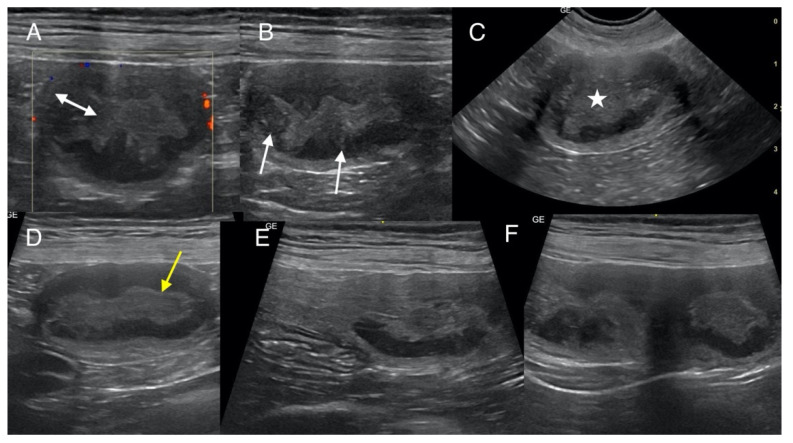
Ultrasound scans of the uterus obtained using a linear probe on the 32nd (**A**,**B**,**D**–**F**) and microconvex probe on the 38th (**C**) day after mating. The examination revealed ampullary dilatation of the uterine horns; transverse sections (**A**–**C**) and sagittal sections (**D**–**F**). Thickening and irregularities of the endometrium are visible (double-headed arrow), as well as few hyperechoic foci and streaks (arrows). The inner layer of the endometrium adjacent to the lumen is hyperechoic (yellow arrow). The presence of hyperechoic fluid content can be seen in the lumen in the thickened part of the uterus (asterisk). Visible vascularization of the uterine wall (**A**). The image of the left horn shows adjacent areas of thickening (**F**) without narrowing, and in the right horn, there is a clear demarcation from the normal part of the horn (**E**).

The owners did not consent to any additional tests, including blood tests, urinalysis, or screening for infectious agents such as *Brucella canis*. A follow-up examination of the uterus, ovaries, and the entire abdominal cavity was performed a week later (32nd day post-mating) by specialists in ultrasonography and reproduction using a microconvex probe (6–10 MHz; GE Healthcare, Chicago, IL, USA) and a linear probe (5–13 MHz; GE Healthcare, Chicago, IL, USA) in B-mode (Versana Premier; GE Healthcare, Chicago, IL, USA). Multiple ampullary dilations in both uterine horns were confirmed. Next, ultrasound evaluation was conducted on the 38th day post-breeding. Again, focal dilations were observed along both uterine horns, manifesting as oval thickening measuring 2.0–2.6 × 3.0–3.6 cm. Six lesions were present in the left uterine horn, four of which were closely connected and poorly separated, one located nearby, and another near the uterine body. Two thickenings were visualized in the right horn, closer to the ovary, separated by an unaffected short segment of uterine tissue. The altered segments exhibited a layered architecture, and the endometrium was noticeably thickened to 0.3–0.7 cm. The mucosal surface appeared wavy, with irregular segmentations and a hyperechoic surface, and demonstrated apparent depressions best visualized in transverse scans performed by linear and microconvex probes (Figure 1). The mucosal layer was hypoechogenic and predominantly homogeneous to slightly heterogeneous; segmentally, irregular hyperechoic points and weakly defined hyperechoic linear bands were seen. A small but variable amount of condensed hyperechoic fluid was seen in the uterine lumen. The segments between the lesions were uniform but only slightly thickened (up to about 1 cm). No endometrial cysts were observed. Neither increased mesenteric reaction nor free fluid was found around the uterine horns. The medial iliac lymph nodes were not enlarged. The Doppler examination showed vascularization originating from the uterine vessels (presumably both veins and arteries) encasing the external surface of the ampullary thickenings (Figure 1A). Both ovaries were enlarged (left: 1.2 × 2.2 cm, right: 1.4 × 2.4 cm), exhibiting characteristics typical of diestrus, with a corrugated surface and the presence of hypoechogenic areas indicative of corpora lutea. In the differential diagnosis, the following conditions were considered: possible embryonic resorption, endometrial inflammatory remodeling, focal hyperplasia with fluid retention, and pseudo-placental proliferation.

**Figure 2:**
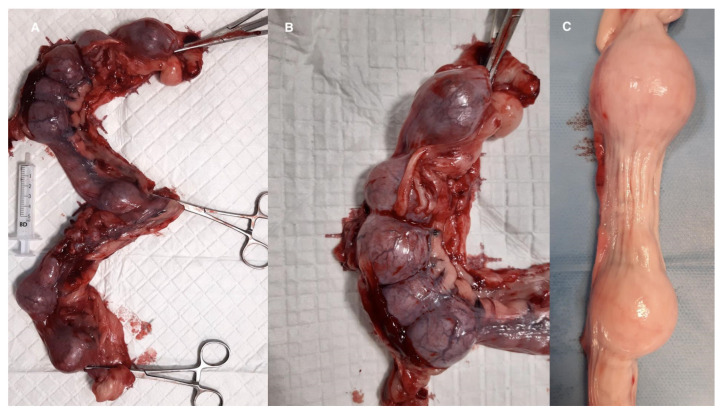
(**A**) The uterine horns following ovariohysterectomy on day 38 after mating. (**B**) Numerous conjoining thickenings in the left horn are present. Uterine horn featuring multiple ampullary swellings, providing a close-up view of the anatomical complexities. (**B**) Left horn; (**C**) right horn.

**Figure 3:**
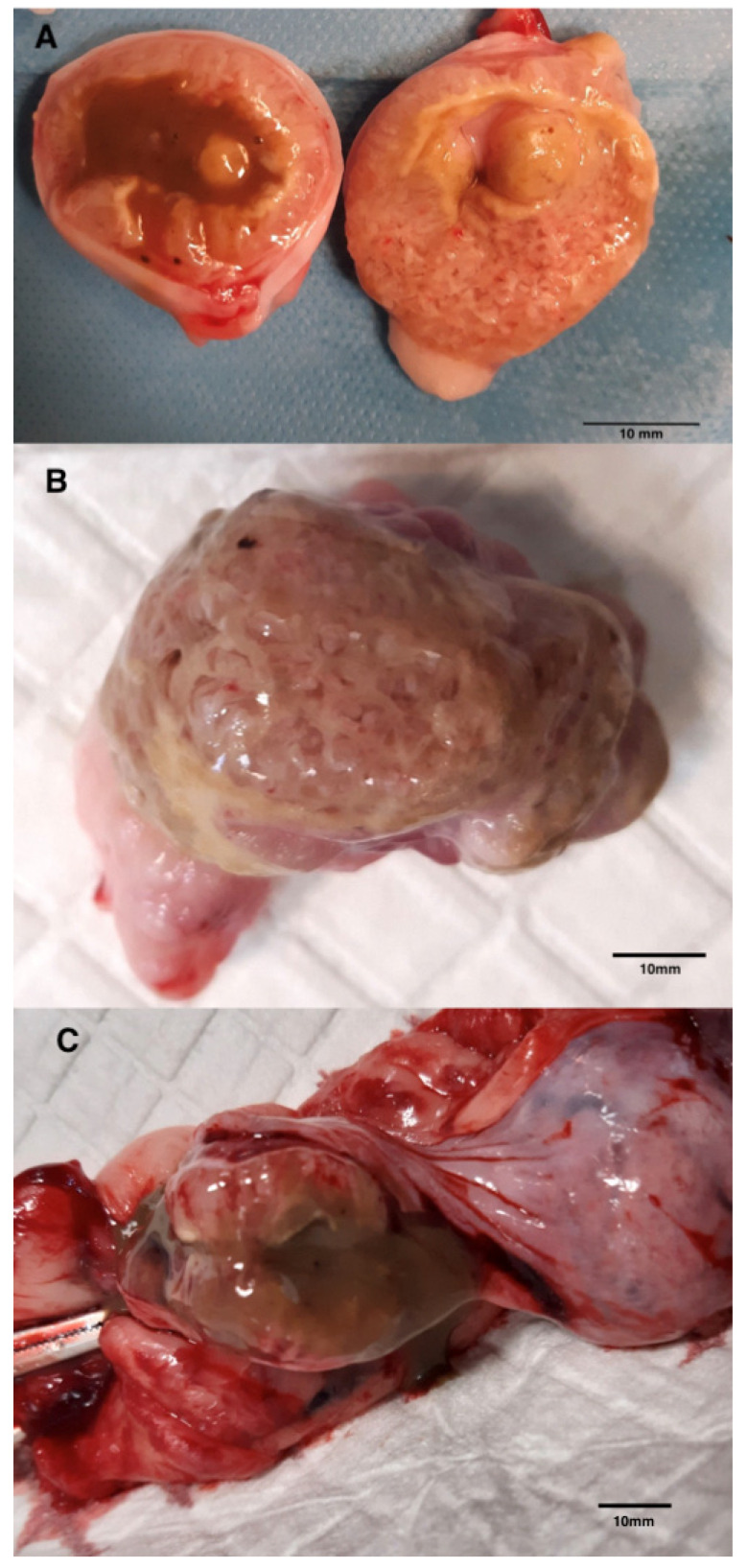
(**A**) Cross-section an ampullary thickened fragment of the uterine horn with visualization of the contents of cloudy, brownish mucous secretion and hyperplastic endometrium. (**B**) Close-up of the internal surface of the mucosa. (**C**) Longitudinal section showing a significant amount of brown secretion.

The bitch underwent an ovariohysterectomy on the 38th day after mating. Upon visual examination, there were distinct, pliable, and spherically shaped masses ranging between approximately 2 cm and 3.6 cm in size in both uterine horns (Figure 2 and Figure 3). The enlargement of the ovaries corresponded with the ultrasonographic findings. A swab was taken for microbiological examination from the fluid present in the uterine cavity. There was no bacterial growth detected, although the antibiotic was discontinued 6 days prior to sample collection.

From a macroscopic perspective, upon longitudinal and transverse sectioning of the thickened uterine horns, a dense, sticky, mucous, turbid, gray-green to brownish fluid content was observed. The endometrial surface appeared dirty pink, villous, and edematous, with segments of smoother yellowish areas. The regions between the lesions did not contain fluid, and the endometrium appeared unchanged.

In the ovaries, multiple corpora lutea were observed. Five thickened fragments of the uterine horns were examined histopathologically in two independent laboratories—two fragments in the first laboratory and three in the second. Specimens were fixed in 10% buffered formaldehyde, subsequently dehydrated through graded ethanol and xylene baths, and then embedded in paraffin wax. Sections of 4 μm thickness were prepared and stained with hematoxylin and eosin (H&E) for examination. The analyses were examined using a standard light microscope Olympus BX41 (Olympus, Tokyo, Japan).

**Figure 4:**
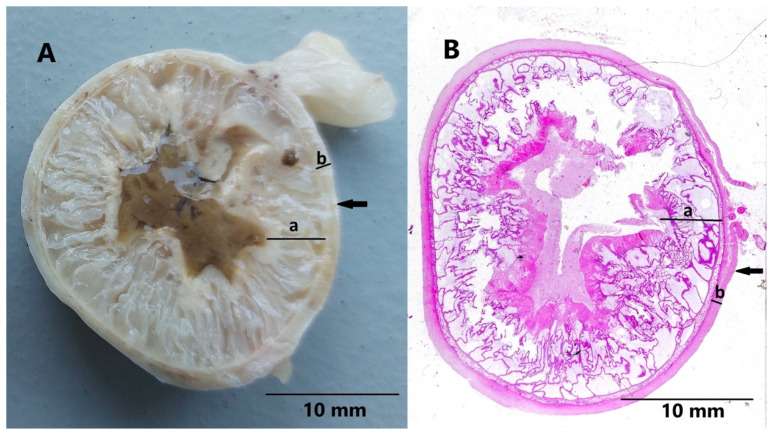
(**A**) Cross-section of the altered segment of uterine horn fixed in formalin. (**B**) Microscopic view of the transverse section of the affected uterine horn segment presenting distension of the uterus with mucosal proliferations forming long projections, and uterine lumen filled with proteinaceous material; moreover, endometrium (a), myometrium (b), and perimetrium (arrow) are visible in both figures; hematoxylin–eosin staining, magnification 10×.

**Figure 5:**
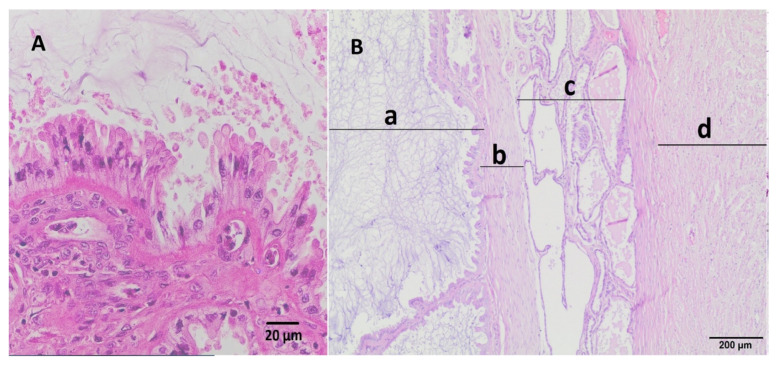
(**A**) Histopathological structure of inner surface of mucosa with uterine mucosal projections covered by a layer of cuboidal or columnar epithelium, uterine lumen is visible at the top of figure; hematoxylin–eosin staining, magnification 40×. (**B**) Microscopic view showing three layers of the uterine mucosa, including (a) villous folds (projections) of the mucosa covered by endometrial epithelium separated by proteinaceous material, (b) a thin band of fibrous connective tissue, (c) distended endometrial glands commonly filled with proteinaceous fluid, and (d) myometrium; hematoxylin–eosin staining, magnification 20×.

In the histopathological examination (Figure 4 and Figure 5), localized extensive, segmental thickening of the uterine wall was observed. Three distinct layers of endometrium were identified. The innermost layer was composed of villous folds lined by endometrial epithelium, separated by mucous material. The middle layer consisted of a thin band of connective tissue covered with epithelial cells, and the outermost layer contained elongated endometrial glands. The final histopathological diagnosis as pseudo-decidual endometrial hyperplasia (PEH) was carried out. There was no microscopic evidence of inflammatory infiltrate or embryonic resorption.

## 3. Discussion

Unlike CEH, PEH is generally a rare uterine pathology [7,12,13,15,17]. It occurs in both young and middle-aged bitches and typically lacks clinical symptoms. In comparison, CEH occurs mainly in older, unsterilized bitches and can lead to pyometra complex. In most CEH cases, the uterus has no ampullar shape, and the cysts are not greater than a few mm in diameter [7,15].

In the presented case, ultrasonographic examination revealed a physiological and morphological stage different from that of a typical pregnancy. Embryos were no detected in either the ultrasound or the histopathological examination. It should be emphasized that histopathological examination was conducted on five out of eight enlarged uterine fragments; however, their macroscopic appearance was similar in all eight cases. Nonetheless, it cannot be definitively ruled out that embryonic tissues might have been present in the remaining three fragments, although this appears highly unlikely. This situation could result from early fetal resorption or anomalous development of placenta-like tissues in the absence of a pregnancy. One of the potential contributing factors could be an infection triggered by the mating process, as described before [12]. In bitches after mating, ultrasonographic differentiation between early embryonic resorption (before day 25) and other conditions such as PEH is challenging, as both processes involve ampullary enlargement of the uterine horn(s), structural remodeling of the uterine wall, and the accumulation of fluid content inside. The only differentiating criteria are longitudinal ultrasound monitoring, where lesions are observed not to undergo involution, followed by histopathological examination of the endometrium [11,12,13]. In our case, the bitch underwent ultrasonographic monitoring for 13 consecutive days, and no changes in the uterine image were observed during this period. Marino described similar findings, including wall thickening with a heterogeneous, slightly echogenic structure, irregularly shaped and resembling cerebral gyri, with well-preserved wall layering in one dog. In another animal, the uterus contained a small amount of poorly fragmented intraluminal fluid and an intraluminal parenchymal proliferation measuring approximately 1.5–2 cm [13]. Ma Ling-Ya et al. reported a circumferential mural thickening in the uterine horn with a distinctly layered appearance [12].

PEH is typically diagnosed as a single lesion, less frequently as a double lesion, and is most observed during the luteal (diestrus) phase when progesterone levels are elevated. In contrast, our case presented with eight thickened lesions in the uterine horns, a number not previously described. However, two or three lesions have been documented [12,13], with a maximum of four reported [13]. As in these publications, we included PEH in the differential diagnosis based on ultrasonographic findings.

PEH can be a spontaneous process [13,17], or through the implantation of foreign substances, agents, or bacteria [8,9,10,11]. Additionally, PEH has been identified in bitches that were mated without resulting in pregnancy [12,13], as well as in uterine where pregnancy developed concurrently [11]. This proliferative abnormality is usually diagnosed in bitches accidentally during ultrasound examination or after ovariohysterectomy. In the presented case, the most probable trigger for the formation of multiple PEH foci was early embryonic resorption or infection following natural mating, as previously described, which may explain the multiplicity of the lesions [12]. However, the exact cause of this condition remains unclear. During an ultrasound examination, distinguishing PEH from the early phase of fetal resorption can be challenging, especially when the examination is performed after a known mating to detect early pregnancy or in a bitch with an unknown history. Embryonic resorption is characterized by the loss of the normal anechoic gestational chamber, the accumulation of echogenic material within the uterine lumen, the absence of embryonic heartbeat, progressive embryonic disintegration, and ultimately, the collapse of the gestational chamber accompanied by thickening of the uterine wall [18,19,20]. According to available data, resorption ampullae in early embryonic resorption (before day 25 of pregnancy) depend on the timing of resorption and are characterized by the presence of hypoechoic rather than anechoic fluid in the yolk sac, echogenic particles within the yolk sac fluid, and later, a reduction in fluid volume (embryonic shrinkage) with internal bulging of the uterine wall. Frequently, the uterus surrounding the embryo appears more hypoechoic compared to the inter-embryonic uterine areas. In most cases, there is a gradual reduction in fluid volume within 2–3 days, and following resorption, the uterus appears homogeneous and moderately hypoechoic, resembling the ultrasonographic appearance of the postpartum uterus [18,19,20]. The reversibility of PEH is not well documented; however, it is suggested that the condition may regress if hormonal influences, particularly progesterone levels, are normalized [12]. Given the dog’s age and the owner’s request to exclude the animal from further breeding, an ovariohysterectomy was performed as a definitive solution.

PEH was further confirmed by morphological and histopathological examination of the uterus. Macroscopically, the appearance was consistent with descriptions in the literature, showing an ampullary dilation of the uterus, resembling an embryonic ampulla around days 30–35 of pregnancy [12,15,17]. It is important to note that the macroscopic appearance of the unopened uterus may resemble pregnancy, focal pyometra, or a regressing placental site [13]. In our case, the macroscopic appearance of the opened uterus differed in color and structure. In the study by Marino et al. (2021), the uterine mucosa was reddish and thickened, with soft whitish-gray tissue and turbid fluid in the lumen [13]. Ling-Ya Ma et al. (2020) described a thickened endometrium with colorless to pale yellow mucoserous fluid [12]. Sato et al. (2011) observed a large amount of semi-transparent, greenish mucoserous fluid in the uterine lumen [17]. Our observations align with the literature, particularly regarding the presence of villous structures on the endometrial surface and fluid in the uterine lumen. Differences in the mucosa’s color structure and the fluid’s composition compared to the reports by Marino, Ling-Ya Ma, and Sato may reflect the stage of the process or specific endometrial reactions in PEH cases [12,13,17].

The histopathological appearance of the observed lesions in our case was consistent with findings reported by Marino et al. (2021), Ling-Ya Ma et al. (2020), and Sato (2011) [12,13,17].

The endometrium was characterized by the presence of three distinct layers that resemble those observed in the pregnant uterus’s endometrium. Additionally, the muscular layer was thinner in the affected areas, compared to the unaffected segments. Moreover, in the innermost layer of the endometrium adjacent to the uterine lumen (described as the spongy layer by Marino et al., 2021), a proliferating population of columnar and cuboidal cells was visible, undergoing degeneration and necrosis, with desquamation into the uterine lumen where they mixed with mucus-like material [13].

The layer described as the labyrinth was absent in our case, similar to some cases of PEH in dogs reported by Marino et al. and in the clinical case described by Sato (2011) [13,17]. Likewise, the labyrinth was also not observed following pharmacologically induced embryonic death in dogs after a few days [21].

Based on literature data, histopathological examination is a valuable tool in differential diagnosis [11,12,13,21]. This type of diagnosis seems definitive in bitches that have not been mated. However, in the case of mated bitches, it appears reasonable that a conclusive diagnosis should be made based on histopathological analysis confirming the characteristic structure of the uterine wall and the absence of embryonic remnants. Assuming that PEH can be induced by fertilization and that early embryonic resorption may result in no detectable embryonic remnants on histopathology, continuous monitoring of lesion persistence seems to be the only way to complement the diagnosis. This was observed in a previously described case of a bitch with concurrent PEH and a carried-to-term pregnancy [11]. Similarly, in our case, the uterine changes did not regress at all over 13 days, remaining completely unchanged, contrary to what would be expected in uterus after embryonic resorption observed in ultrasound [18,19,20].

## 4. Conclusions

The presented clinical case is unusual due to the presence of multiple PEH sites, as single PEH lesions are more commonly observed. To date, PEH with such extensive and numerous lesions has not been previously reported. PEH has also not been reported in this breed.

## Data Availability

Data are available on request from the corresponding author.
